# Risk of Liver Injury Associated with Chinese Herbal Products Containing *Radix bupleuri* in 639,779 Patients with Hepatitis B Virus Infection

**DOI:** 10.1371/journal.pone.0016064

**Published:** 2011-01-12

**Authors:** Chang-Hsing Lee, Jung-Der Wang, Pau-Chung Chen

**Affiliations:** 1 Institute of Occupational Medicine and Industrial Hygiene, National Taiwan University College of Public Health, Taipei, Taiwan; 2 Department of Occupational Medicine, Ton Yen General Hospital, Hsin-Chu, Taiwan; 3 Department of Public Health, National Cheng Kung University College of Medicine, Tainan, Taiwan; 4 Department of Environmental and Occupational Medicine, National Taiwan University Hospital, Taipei, Taiwan; The University of Hong Kong, Hong Kong

## Abstract

**Background:**

Chinese herbal products (CHPs) containing *Radix bupleuri* are often prescribed for chronic hepatitis. There have been no epidemiological studies in populations with hepatitis B virus (HBV) infection. Our study was conducted to determine the association between the use of CHPs containing *Radix bupleuri* and the risk of hospitalisation related to liver injury among HBV-infected patients in Taiwan.

**Methods:**

From a total of 639,779 patients with diagnoses related to HBV infection, we included hospitalised adult cases with a primary diagnosis of liver injury in the database of Taiwan's national health insurance during the period 1997–2004. Case-control and case-crossover designs were used to assess the risk of hospitalisation with conditional logistic regression models constructed and adjusted for 270 conventionally hepatotoxic drugs. Cumulative doses of these CHPs and *Radix bupleuri* were assessed for any dose-response relationship.

**Findings:**

In total, we collected 1,080 cases fulfilled the inclusion criteria. In the case-control design, the adjusted odds ratio was 1.90 (95% confidence interval [CI]: 1.30 to 2.77). The risks from prescribing the CHPs Xiao-Chai-Hu-Tang and Long-Dan-Xie-Gan-Tang were significantly high, and dose-response relationships were found. The risk of adding each 19 gm dose of *Radix bupleuri* was 2.19 (95% CI: 1.66 to 2.89). The results using the case-crossover design remained similar.

**Conclusions:**

Prescribing Xiao-Chai-Hu-Tang, Long-Dan-Xie-Gan-Tang, or CHPs containing more than 19 gram of *Radix bupleuri* in HBV-infected patients might increase their risks of liver injury. Further studies are indicated to corroborate the above findings.

## Introduction

An increasing number of herbal remedies are now being reported as hepatotoxic, with such reports on Chinese herbal products (CHPs) recently including a variety of types, such as *Radix scutellariae*, *Radix bupleuri*,[1] *Herba ephedrae*,[2] *Radix polygoni multiflori*,[3] *Atractylodis macrocephalae rhizoma*, *Radix glycyrrhizae*,[4] *Radix paeoniae*, *Cortex moutan*, and *Cortex dictamni.* [5], [6] However, regular users seem still to regard these remedies as ‘natural’ and nontoxic.[7] CHPs are also prescribed for chronic hepatitis[8] in Taiwan, especially in patients with hepatitis B virus (HBV) infection.[9]

CHPs containing *Radix bupleuri*, such as Da-Chai-Hu-Tang (Dai-saiko-to, TJ-8) and Xiao-Chai-Hu-Tang (syo-saiko-to, TJ-9) have been reported to induce hepatotoxicity.[1], [10], [11], [12] Among them, Xiao-Chai-Hu-Tang is often prescribed to treat HBV infection and the usual posology for Radix bupleuri is doses of 3–9 g/day. [8], [13], [14] However, another study undertaken in Hong Kong found that 32% of the patients with HBV infection had used CHPs, and 9% of them experienced moderate to severe side effects.[15]

Drug- or herb-induced liver injury is usually difficult to detect.[16], [17] A post-market pharmacoepidemiologic survey in a large automated database provides a method to detect the risk associated with herbal medications. The National Health Insurance (NHI) program in Taiwan is a universal system of compulsory health insurance that has been providing coverage for 96.2% of the population of Taiwan since the end of 2000. The Taiwanese government reimburses not only general healthcare expenditures but also the costs of prescriptions for CHPs (amounting to 780 different kinds of single herbs and mixed formulae in concentrated extract form). [18] This computerised database of CHPs provides us with an invaluable opportunity to undertake a population-based study.

This study was conducted to determine the association between the use of CHPs containing *Radix bupleuri* and the risk of hospitalisation related to liver injury among HBV-infected patients in Taiwan.

## Methods

### Ethics Statement

With strict confidentiality guidelines being closely followed in accordance with personal electronic data protection regulations, The National Health Research Institutes of Taiwan anonym zed and maintained the NHI reimbursement data as files suitable for research.[19] The identification numbers of all individuals with reimbursement data in the NHI database were encrypted to protect the privacy of the individuals. In addition, this study was also approved by the Ethics Review Board at the National Taiwan University College of Public Health.

### NHI databases in Taiwan

The dataset for the study was obtained from the NHI Research Database, which is a nationwide, population-based, reimbursement database and all the personal identification has been encrypted for research use.[20] The files are comprised of comprehensive information on all medications prescribed to all insured individuals. We conducted this study on both outpatient visits and inpatient admission databases from January 1, 1997 through December 31, 2004.

### Case selection

Cases included hospitalised patients who were older than 18 years of age and who suffered from liver injuries with diagnoses related to HBV infection coded as 070.2, 070.3 and V02.61 by the International Classification of Diseases, 9th Revision (ICD-9).[21] Because liver diseases were the important health problems for our country, the government pay more intention for this health issue and make the guideline for diagnosis. [22] However, to prevent any case misclassification, we only included incident cases with a primary diagnosis of liver injury, and we excluded cases with other diagnoses on admission or cases reported only from outpatient clinics. Primary diagnoses of liver injury coded by the ICD-9 included acute and sub-acute necrosis of the liver (570), toxic hepatitis (573.3), other specified disorders of liver (573.8), and unspecified disorders of liver (573.9). Moreover, we excluded patients who had been diagnosed with the following conditions at any time before admission: viral hepatitis A, viral hepatitis C, other viral hepatitis (070.0, 070.1, 070.4, 070.5, 070.6, 070.7, and 070.9), and viral hepatitis carriers (V02.60, V02.62, and V02.69); cytomegalovirus and coxsackie virus diseases and infectious mononucleosis (573.1 to 573.2); cholelithiasis (574.0 to 574.9); alcoholic liver diseases, chronic hepatitis, cirrhosis of liver without mention of alcohol, biliary cirrhosis, Other chronic nonalcoholic liver disease, unspecified chronic liver disease without mention of alcohol (571.0 to 571.9); abscess of the liver, portal pyemia, hepatic coma, portal hypertension, hepatorenal syndrome, and chronic passive congestion of the liver (572.0 to 573.0); malignant neoplasm of liver and intrahepatic bile ducts (155.0 to 155.2), liver metastasis (197.7), carcinoma in situ of the liver and biliary system (230.8); and liver disorders during pregnancy (646.7).

### Selection of referents in the case-control and case-crossover designs

First, we analysed the datasets using a case-control design. Four control subjects or referents for each case were randomly selected by matching the age and gender from the same population with HBV infection who did not have previous diagnoses of either liver injury or any of the diseases that met the exclusion criteria for cases. Namely, we first included all people with HBV-infected diagnosis, excluded those with aforementioned diagnoses, and then randomly drew 4 age- and gender- matched controls for each case. In the case-crossover design, we set four referent exposure windows before the recent exposure window and set 90 days between them to prevent any carryover effect. We applied the 30-day exposure windows in both designs.

### CHPs containing *Radix bupleuri* and covariates

We selected all CHPs containing *Radix bupleuri* as our target drugs. We then calculated the cumulative dose of CHPs prescribed during each exposure window. Otherwise, we estimated the crude dosage of *Radix bupleuri* in the CHPs in terms of both weight and concentrated proportions, based on the concentrated mixed products manufactured by different CHP companies and translated to the dose of crude herbs by concentrated ratio. Within a commercial brand of Xiao-Chai-Hu-Tang, for example, the registered weight proportion of *Radix bupleuri* was 0.33, whilst the concentrated proportion was 4 and added the equal weight starch; thus, a final product containing 1.0 gm of such a concentrated CHP would be equivalent to 0.67 gm of *Radix bupleuri* in crude form. The cumulative dosage of *Radix bupleuri* within the CHPs was also calculated by adding together the total dosage prescribed during each exposure windows. Then we stratified with the median dose of the cumulative dosages to test for trends of dose-response. Other hepatotoxic drugs and co-morbidities were considered as covariates in the models. We undertook a search of the Micromedex® database [23] for a total of 702 generic drugs that had been previously reported as having any connection with hepatotoxicity. The NHI in Taiwan regularly reimbursed for 270 of these generic drugs. We calculated the scores of Charlson Comorbidity Index by using ICD-9 codes to determine the condition of 1-year comorbidity.[24]

### Data analysis

The use of CHPs containing *Radix bupleuri* by each case subject during the exposure window was contrasted with the use of the same CHPs for the same duration by the four matched control subjects. The odds ratio (OR) was calculated for the exposure-odds of case subjects and control subjects and denoted as a case-control estimate. In the case-crossover design, the prevalence of CHPs containing *Radix bupleuri* during the single recent exposure window was contrasted with the prevalence over four reference windows for the same case subject. Because the study designs included one case matched with four controls or one recent exposure window matched with four reference exposure windows, we analysed the data through a conditional logistic regression model to explore the association between hospitalisation and CHPs containing *Radix bupleuri*. By adjusting two covariates in the case-control design (the Charlson co-morbidity index scores and the frequency of the time-variant hepatotoxic drugs during each exposure window) and by adjusting the latter in the case-crossover design, we obtained the adjusted ORs. The data analysis was performed and modelled to calculate ORs and 95% CIs using SAS version 9.13 software (SAS Institute Inc., Cary, NC).

## Results

After conforming to the inclusion and exclusion criteria from the population with 639,779 patients with HBV infection, the sample was comprised of 1,080 cases with at least one 30-day referent exposure window during the study period. Among them, 13.4% were 60 year of age or older, with a mean age of 40.2±14.6 years. Table 1 shows that case subjects had larger Charlson co-morbidity index scores and more pre-admission use of hepatotoxic drugs and CHPs per person than control subjects had.

**Table 1:** Characteristics, co-morbidities, prescriptions and time-dependent hepatotoxic drugs for study subjects with viral hepatitis B infection, 1997–2004.

	Case		Control	
Characteristics	Nos.	%	Nos.	%
Total	1,080	100.0	4,320	100.0
Time independent covariates				
Sex				
Male	781	72.4	3124	72.4
Female	299	27.6	1196	27.6
Age				
19–29 years	297	27.5	1188	27.5
29–39 years	292	27.1	1168	27.1
39–49 years	201	18.6	804	18.6
49–59 years	145	13.4	580	13.4
> = 60 years	145	13.4	580	13.4
Admission diagnosis (ICD-9 code)				
Acute and sub-acute liver necrosis (570)	792	73.3	0	0
Toxic hepatitis (573.3)	210	19.5	0	0
Other specified liver disorders (573.8)	65	6.0	0	0
Unspecified liver disorders (573.9)	13	1.2	0	0
Charlson co-morbidity index before admission				
0	281	26.0	2756	63.8
1–2	639	59.2	1334	30.9
3–5	135	12.5	212	4.9
>5	25	2.3	18	0.4
Co-morbidities may enhance susceptibility to drug-induced liver injury	No.^a^	%	No.^a^	%
Diabetes mellitus	138	12.8	432	10.0
Obesity and hyperlipidemia	126	11.7	605	14.0
Neoplasms	94	8.7	729	16.9
Essential hypertension	85	7.9	501	11.6
Chronic kidney disease and renal failure	38	3.5	143	3.3
Hyperthyroidism	15	1.4	80	1.9
Systemic lupus erythematosus	7	0.6	10	0.2
Fasting, malnutrition	6	0.6	10	0.2
Pregnancy	0	0.0	45	1.0
Time dependent covariates				
Prescriptions before admission	No.^b^	per person	No.^b^	per person
Chinese herbal products with *Radix bupleuri*	3,577	3.3	6,836	1.6
Hepatotoxic drugs	121,528	112.5	350,605	81.2

ICD-9, International Classification of Diseases, 9th Revision.

a Each subject could have more than one co-morbidity before admission.

b Each subject could have more than one prescription and co-prescription.

In the case-control design, the adjusted OR during the 30-day window was 1.90 (95% Confidence Interval (CI): 1.30 to 2.77) between all products with *Radix bupleuri* and admissions with liver injury. The risks from Xiao-Chai-Hu-Tang and Long-Dan-Xie-Gan-Tang were significantly high, and the tests for trends in the dose-response reaction were significant. The additional risk incurred by adding each 19 gm dose of *Radix bupleuri* was 2.19 (CI: 1.66 to 2.89). The results using the case-crossover design remained similar.

## Discussion

Although our study found that the prescription of two CHPs containing *Radix bupleuri*, Xiao-Chai-Hu-Tang and Long-Dan-Xie-Gan-Tang, increased the risk of hospitalisation for liver injury among HBV-infected subjects, it does not necessarily follow that the association might be causal. We have following arguments to suspect that the hypothesis is plausible.

The risk trends were consistent in both designs—case-control and case crossover, and there seems a dose-response relationship, as summarized in the Table 2. To prevent potential confounding by misdiagnosis, we deliberately included only patients who were hospitalised and excluded other possible causes of hepatobiliary diseases, including all other hepatitis-related infections, alcohol-related liver disorders, cholelithiasis and de-compensated hepatic conditions, as these conditions might make patients more likely to suffer liver injury from exposure to potentially hepatotoxic drugs. Our estimates were more conservative as we did not include the above cases. Because we did not have any direct access to the original clinical data, our study was necessarily limited to the more severe cases resulting in hospitalisations, which may increase the validity of the diagnosis but undoubtedly resulted in an underestimation of hepatotoxicity with only mild manifestations.

**Table 2:** Number of exposed subjects with viral hepatitis B in the 30-day windows and adjusted odds ratios between hospitalisations with liver injury and Chinese herbal products with *Radix bupleuri* by case-control and case-crossover designs, 1997–2004.

Chinese herbal products (CHPs)	Exposed no. of cases in recent window	Exposed no. of controls in recent windows	Case-control design			Exposed no. of cases in reference windows	Case-crossover design		
			OR^a^	95% C.I.			OR^b^	95% C.I.	
Total no.	1,080	4,320				4,320			
All products c with *Radix bupleuri*	61	89	1.90	1.30	2.77	155	1.75	1.22	2.50
Xiao-Chai-Hu-Tang	19	23	2.91	1.36	6.24	26	2.81	1.36	5.78
Cumulative dose > = 31 gm d	8	11	1.92	0.69	5.36	15	1.63	0.60	4.45
Cumulative dose <31 gm	11	12	3.08	1.18	8.09	11	3.43	1.37	8.59
Test of linear trend for 0, <31, > = 31 gm			1.77	1.09	2.88		1.67	1.06	2.65
Long-Dan-Xie-Gan-Tang	14	20	3.52	1.56	7.95	29	2.31	1.11	4.80
Cumulative dose > = 36 gm d	8	6	5.14	1.62	16.33	14	2.72	1.01	7.29
Cumulative dose <36 gm	6	14	2.03	0.64	6.44	15	1.69	0.61	4.63
Test of linear trend for 0, <36, > = 36 gm			2.34	1.37	3.99		1.72	1.09	2.73
Jia-Wei-Xia-Yao-San	7	25	2.07	0.72	5.92	26	1.87	0.69	5.06
All other CHPs combined e	36	108	1.42	0.85	2.37	120	1.19	0.76	1.85
Content of *Radix bupleuri* in all CHPs c									
Cumulative dose > = 19 gm d	28	67	5.17	2.79	9.56	81	1.91	1.19	3.06
Cumulative dose <19 gm	33	22	1.40	0.83	2.35	26	1.36	0.85	2.19
Test of linear trend for 0, <19, > = 19 gm			2.19	1.66	2.89		1.44	1.15	1.80

no., number; OR, odds ratio; CI, confidence interval.

a Adjusted for the frequency of the time-variant hepatotoxic drugs during every exposure window and the scores of the Charlson co-morbidity index score for one year prior to admission.

b Adjusted for the frequency of the time-variant hepatotoxic drugs during every exposure window.

c CHPs with *Radix bupleuri* prescribed by cases and controls or in recent and reference windows: Jia-Wei-Xia-Yao-San, Long-Dan-Xie-Gan-Tang, Xiao-Chai-Hu-Tang (Sho-Saiko-To, TJ-9), Chai-Hu-Qing-Gan-Tang, Chai-Hu-Su-Gan-Tang, Jing-Fang-Bai-Du-San, Hsieh-Fu-Chu-Yu-Tang, Chai-Ge-Jie-Ji-Tang, Bu-Zhong-Yi-Qi-Tang, Chai-Hu-Gei-Zhi-Tang, Xia-Yao-San, Zi-Shen-Tong-Er-Tang, Pu-Ji-Xiao-Du-Yin, Qin-Jiao-Bie-Jia-San, Yi- Gan-San, Si-Ni-San, Da-Chai-Hu-Tang, Chai-Hu-Jia-Long-Gu-mu-Li-Tang, Chai-Hu-Xian-Xiong-Tang, Qing-Kong-Gao, San-Zhong-Kui-Jian-Tang, Jing-Jie-Lian-Qiao-Tang, Wan-Dai-Tang, Shen-Mi-Tang, Ren-Shen-Bai-Du-San, Sheng-Yang-Yi-Wei-Tang, Fu-Yuan-Huo-Xue-Tang, Dun-Sou-Tang, and Chai-Hu.

d The cut-off points for categories were the medians of all cumulative doses.

e Cumulative doses cannot be classified due to different CHPs.

Another potential explanation is the confounding by indication, as the two major CHPs were frequently prescribed by Chinese medicine doctors to treat hepatitis. However, the most frequently prescribed CHP containing *Radix bupleuri* for subjects with HBV infection was Jia-Wei-Xia-Yao-San and the adjusted OR was 1.87 (CI: 0.69 to 5.06) by case-crossover design (Table 2). Moreover, prescriptions of all the other CHPs containing *Radix bupleuri* combined together did not show a significantly higher risk of liver injury, either. As these products were usually prescribed to HBV-infected patients, or, supposedly to be associated with HBV infection, the results of no existing association in other CHPs might serve as a contrast against the possibility of confounding by indication. However, since the number for each individual CHPs is small, we cannot draw a strong inference.

To test the potential dose-response relationship, we summed up the cumulative prescribed doses of *Radix bupleuri* for every window period of each patient, and then stratified the groups into 0, below and above the median dose of 19 gm. We detected a statistically significant linear trend between the increased dose and hospitalization related to liver injury (Table 2). We conducted further sensitivity analyses by stratification to clarify the misclassifications and potential confounders in the case-crossover design. The results reveal no significant changes in the ORs of the subgroups stratified by different prescribing conditions, matched patterns and co-morbidities.[16] Although the medications being studied were co-prescribed with seven potentially hepatotoxic drugs, the results reveal no significant contributions of drug-drug interactions. (Appendix S1) Since there is no other alternative explanation for the reasons of hospitalisation, we tentatively conclude that the association between liver injury and CHPs containing *Radix bupleuri* in HBV-infected patients exists and deserves our attention.

In Japan, Xiao-Chai-Hu-Tang and other CHPs containing *Radix bupleuri* produced no significant effect of acute and chronic toxicity on rats.[25] However, these animals were not suffering from HBV infection. With a retrospective study of reviewing HBV-infected cases with liver injury, Yuen et al. found 45 cases with suspected CHP-associated liver injury. [26] A hospital-based questionnaire survey found 10 of 116 patients who had taken CHPs experienced side effects.[15] But they did not explore the detailed active ingredients in the exposed CHPs. The only one clinical trial that involved applying Xiao-Chai-Hu-Tang in 16 HBV-infected patients with cirrhosis for 60 months (or, a total of 80 person years) did not detect any side effect.[27] Our study is conducted on a nation-wide basis and followed for 7 years, of which we collected 281,982 person-years of exposure to Xiao-Chai-Hu-Tang and there were a total of 224 cases with liver injury that required hospitalisation, which reveals an annual incidence rate of 7.94×10^−4^ per year, while that of Long-Dan-Xie-Gan-Tang is 5.96×10^−4^.

Potential limitations of this study, including unmeasured confounders, use of other out-of-pocket drugs, and patient compliance should also be discussed. First, the NHI reimburses for prescribed CHPs in concentrated extract form only, whereas herbal stores can sell raw herbs for patients to self-prepare decoction, according to the Pharmaceutical Affairs Law in Taiwan. The potential impact of current practices should not be ignored. However, as self-purchased raw herbs were not regularly reimbursed, people usually take them once in a while or when the CHPs fails to improve the symptom. Thus, the cumulative dose is usually small after stratification for different window periods. Furthermore, if such habits among patients remained unchanged within a 2-year period, the case-crossover design might eliminate this potential confounding, taking the advantage of each patient serving as his/her own control. Finally, this study also presumed that all prescribed medications were actually taken by subjects as prescribed, which may overestimate the actual ingested dosage, as some degree of noncompliance is always expected.

### Conclusions

Our study found that HBV-infected subjects using Xiao-Chai-Hu-Tang and Long-Dan-Xie-Gan-Tang had an increased risk of liver injury, and there was a statistically significant linear trend of dose-response for the cumulative prescribed doses of *Radix bupleuri*. Thus, we recommend that physicians should carefully monitor hepatic function in the subjects who are taking these CHPs regularly. Moreover, if liver injury occurs, Xiao-Chai-Hu-Tang and Long-Dan-Xie-Gan-Tang should be considered as the potential sources of hepatotoxicity. However, further mechanistic research on the hepatotoxicity of *Radix bupleuri* in the presence of HBV infection is warranted.

## Supporting Information

Appendix S1 Sensitivity analysis of adjusted odds ratios between hospitalisations with liver injury and Xiao-Chai-Hu-Tang and Long-Dan-Xie-Gan-Tang stratified by subgroups of matched patterns, prescribing conditions, co-morbidities and co-prescriptions by case-crossover design, 1997–2004.
(DOC)
