# Supplementary material for: Risk of Liver Injury Associated with Chinese Herbal Products Containing Radix bupleuri in 639,779 Patients with Hepatitis B Virus Infection
Source: PLoS One. 2011 Jan 12;6(1):e16064. doi: 10.1371/journal.pone.0016064 (PMC3020221; doi:10.1371/journal.pone.0016064)
Supplement: Appendix S1 — Sensitivity analysis of adjusted odds ratios between hospitalisations with liver injury and Xiao-Chai-Hu-Tang and Long-Dan-Xie-Gan-Tang stratified by subgroups of matched patterns, prescribing conditions, co-morbidities and co-prescriptions by case-crossover design, 1997–2004. (DOC) [file pone.0016064.s001.doc]

Appendix S1. Sensitivity analysis of adjusted odds ratios between hospitalisations with liver injury and Xiao-Chai-Hu-Tang and Long-Dan-Xie-Gan-Tang stratified by subgroups of matched patterns, prescribing conditions, co-morbidities and co-prescriptions by case-crossover design, 1997-2004

| Models | Items | Xiao-Chai-Hu-Tang | | | | | Long-Dan-Xie-Gan-Tang | | | | |
| --- | --- | --- | --- | --- | --- | --- | --- | --- | --- | --- | --- |
|  |  | Exposed no. of cases in recent window | Exposed no. of controls in recent windows | OR^a^ | 95% CI | | Exposed no. of cases in recent window | Exposed no. of controls in recent windows | OR^a^ | 95% CI | |
|  | Total no. | 1,080 | 4,320 |  |  |  | 1,080 | 4,320 |  |  |  |
| **Main model** | Total population | 19 | 26 | 2.81 | 1.36 | 5.78 | 14 | 29 | 2.31 | 1.11 | 4.80 |
| **Time effects** |  | | | | | |  | | | | |
| Different exposure window patterns | 90-day | 35 | 41 | 4.38 | 2.33 | 8.23 | 29 | 68 | 1.70 | 0.94 | 3.10 |
|  | 60-day | 28 | 39 | 4.26 | 2.23 | 8.14 | 22 | 44 | 2.78 | 1.35 | 5.73 |
|  | 14-day | 15 | 11 | 19.64 | 4.97 | 77.50 | 11 | 16 | 4.74 | 1.79 | 12.57 |
|  | 7-day | 11 | 5 | 9.94 | 2.62 | 37.73 | 7 | 9 | 4.27 | 1.36 | 13.42 |
| **Subgroup effects** |  | | | | | |  | | | | |
| Matched patterns | One risk to two reference windows | 0 | 0 | - | - | - | 0 | 0 | - | - | - |
|  | One risk to three reference windows | 1 | 1 | - | - | - | 0 | 0 | - | - | - |
|  | One risk to four reference windows | 18 | 25 | 2.74 | 1.32 | 5.70 | 14 | 29 | 2.31 | 1.11 | 4.80 |
| Prescribing conditions | Stop formula after admission | 12 | 1 | - | - | - | 10 | 1 | - | - | - |
|  | Die after admission | 1 | 0 | - | - | - | 0 | 1 | - | - |  |
| Co-morbidities may affect hepatotoxicity | Neoplasms | 2 | 2 | 4.00 | 0.25 | 63.95 | 1 | 4 | 4.00 | 0.25 | 63.95 |
|  | Essential hypertension | 0 | 3 | - | - | - | 0 | 2 | - | - | - |
|  | Diabetes mellitus | 0 | 6 | - | - | - | 1 | 4 | - | - | - |
|  | Obesity and hyperlipidemia | 0 | 5 | - | - | - | 1 | 2 | 2.16 | 0.11 | 43.43 |
| Potential hepatotoxic co-prescriptions | Sulfamethoxazole | 14 | 21 | 2.22 | 0.94 | 5.27 | 10 | 14 | 4.23 | 1.59 | 11.31 |
|  | Chlorzoxazone | 13 | 17 | 2.39 | 0.94 | 6.06 | 8 | 19 | 2.20 | 0.84 | 5.76 |
|  | Amlodipine | 0 | 4 | - | - | - | 1 | 2 | - | - | - |
|  | Allopurinol | 0 | 1 | - | - | - | 1 | 2 | - | - | - |
|  | Metformin | 0 | 2 | - | - | - | 0 | 1 | - | - | - |
|  | Rifampin | 2 | 2 | - | - | - | 1 | 0 | - | - | - |
|  | Isoniazid | 0 | 0 | - | - | - | 0 | 0 | - | - | - |

no., number; OR, odds ratio; CI, confidence interval.

^a^ Adjusted for hepatotoxic medications, co- medications with Chinese herbs and hepatotoxic drugs during the exposure windows.
